# Supplementary figures and images for: Automated and Accurate Estimation of Gene Family Abundance from Shotgun Metagenomes
Source: PLoS Comput Biol. 2015 Nov 13;11(11):e1004573. doi: 10.1371/journal.pcbi.1004573 (PMC4643905; doi:10.1371/journal.pcbi.1004573)

Genome Relative Abundance

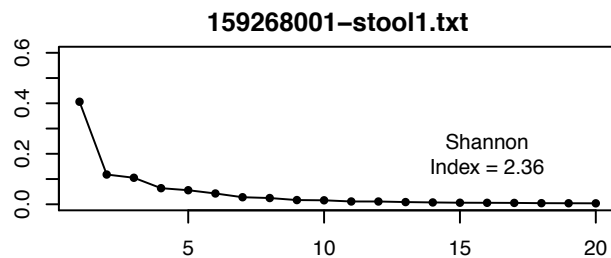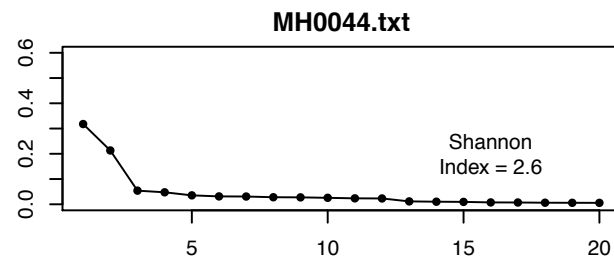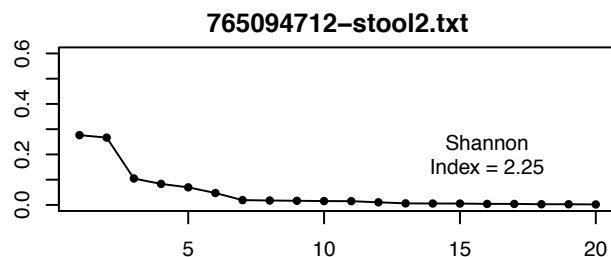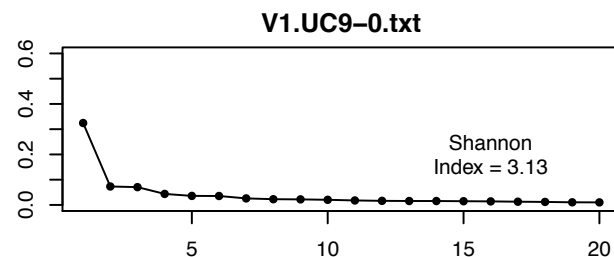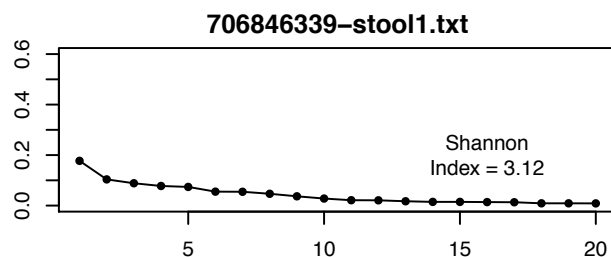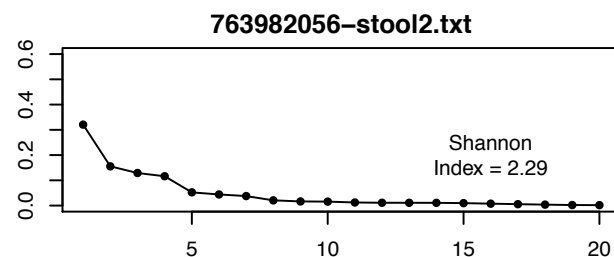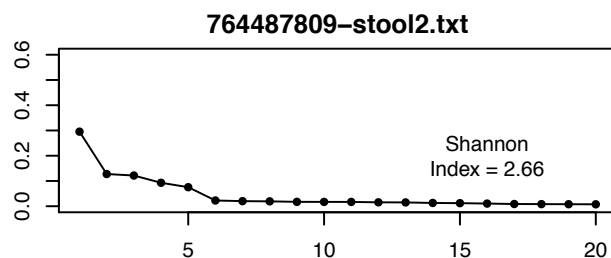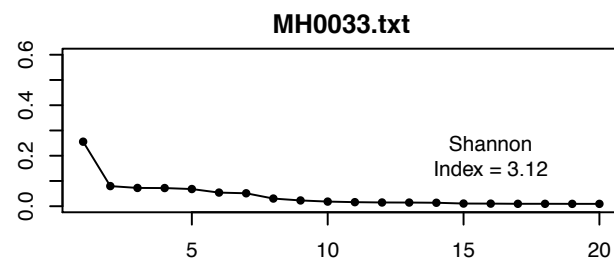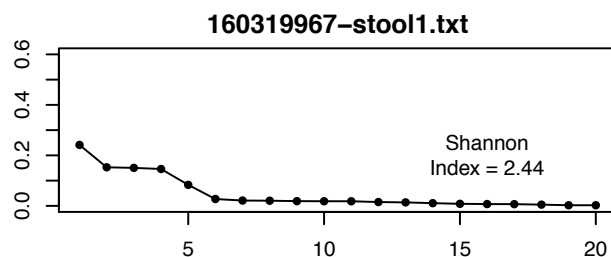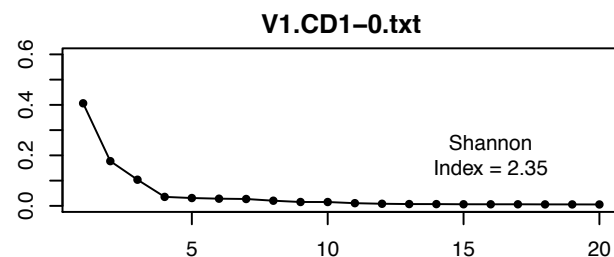

Rank (top 20 only)

Rank (top 20 only)

Supplement: S1 Fig — Relative abundances distributions for the top 20 most abundant species (out of 909 total) in each of the mock communities. Each community contains at least 500 species. (PDF) [file pcbi.1004573.s005.pdf]

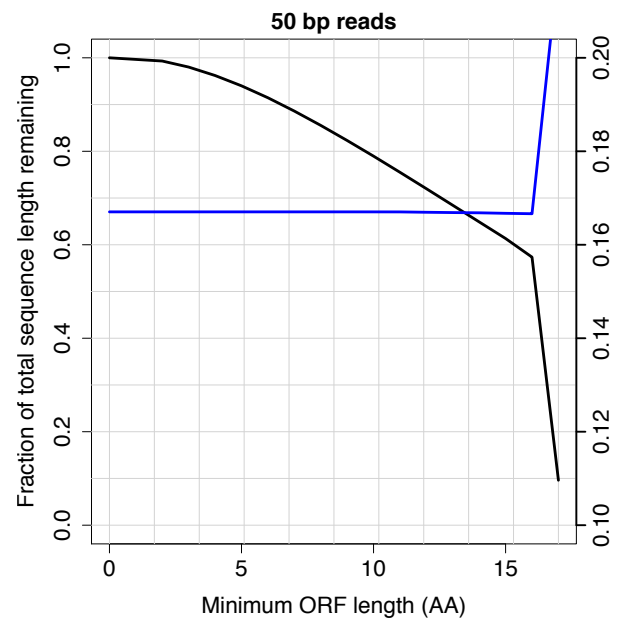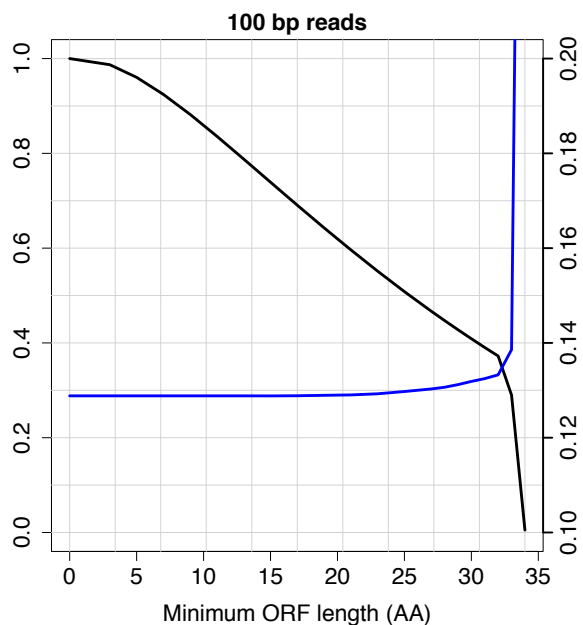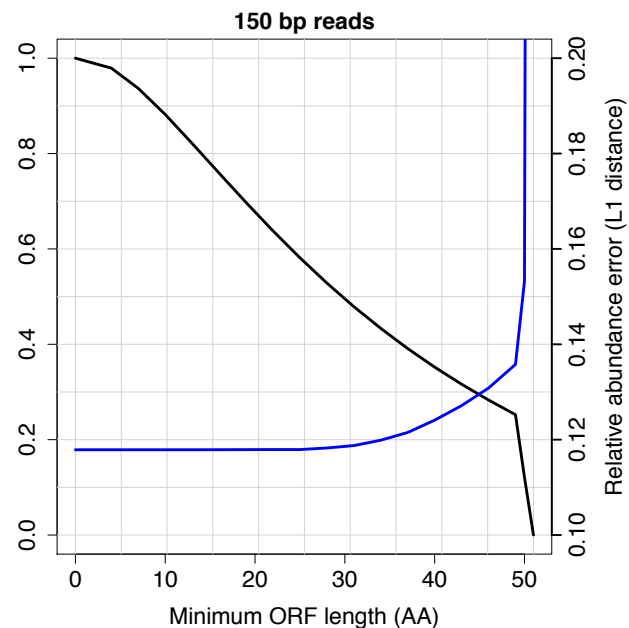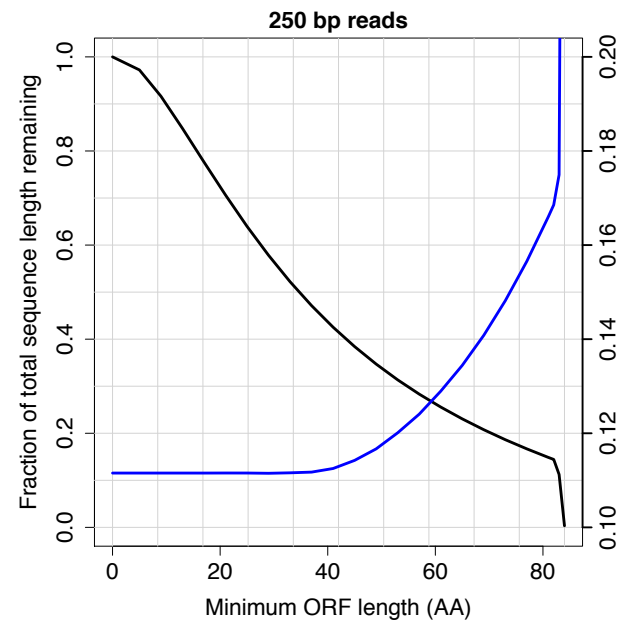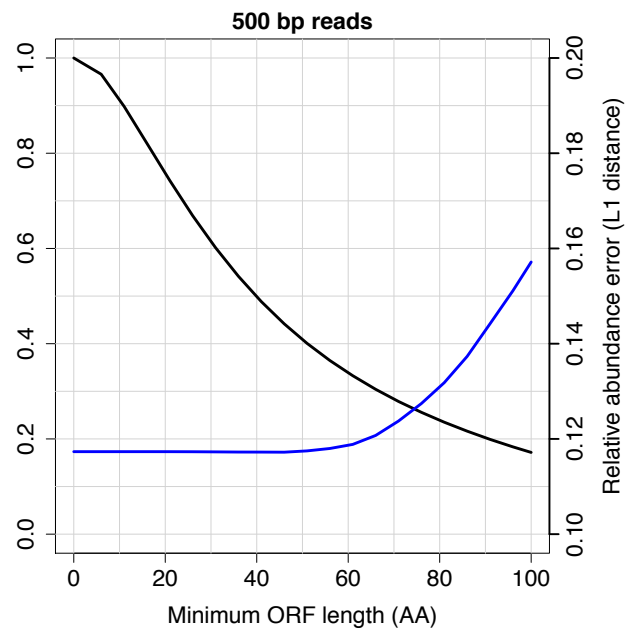

## LEGEND

- Relative abundance error (L1 distance)
- Fraction of total sequence length remaining

Supplement: S2 Fig — Reads from simulated metagenomes (50–500 bp, 1% error rate, 1.2 million reads) were translated into all six possible reading frames and each reading frame was split into multiple peptides at stop codon positions. Peptides were filtered by their sequence length, and the remaining peptides were used to estimate the abundance of protein families in each metagenome. Each plot shows the results for a different metagenome (50, 100, 150, 250 and 500 bp reads). In each plot, the x-axis indicates the minimum length of peptides that were retained and used for homology interference and protein family abundance estimation. For example, a minimum ORF length of 40 indicates that peptides with less than 40 amino acids were discarded. The left y-axis and black curve indicate the fraction of total sequence length (in amino acids) that was left after filtering. The right y-axis and blue curve indicates the error in the resulting protein family abundance estimates. (PDF) [file pcbi.1004573.s006.pdf]

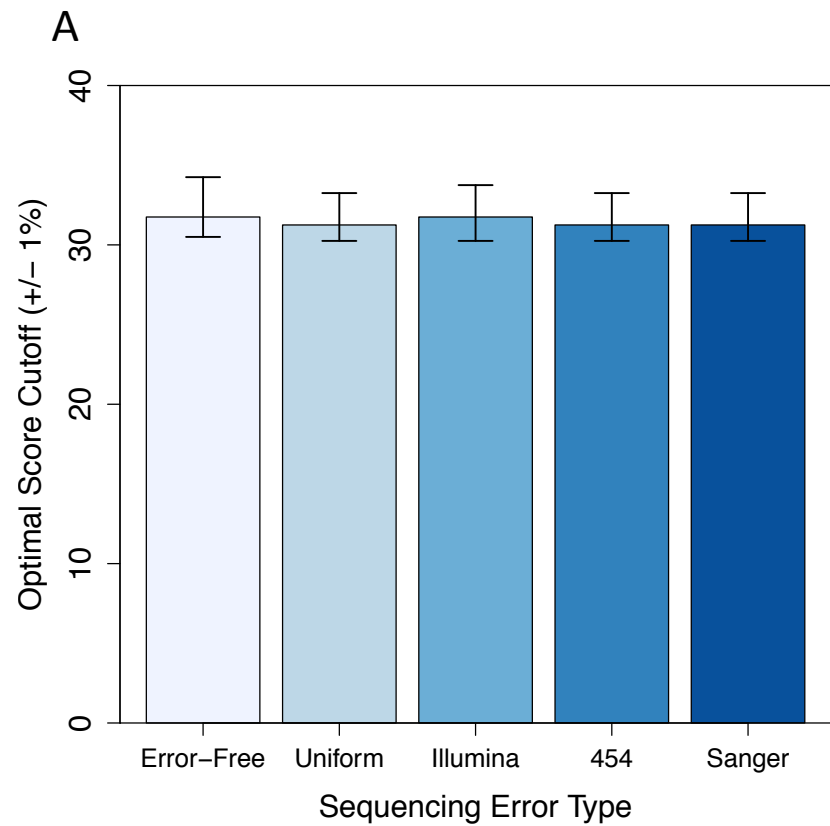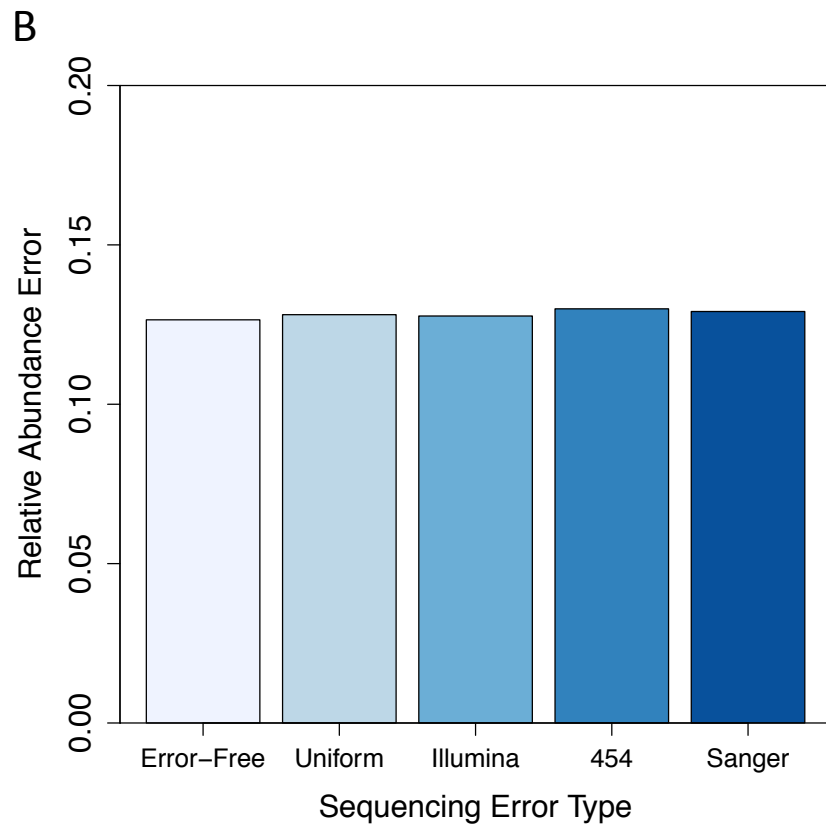

Supplement: S3 Fig — False positive rates for synthetic negatives. 500,000 reads were randomly sampled from community 160319967-stool1 at various read lengths (50, 100, 150, 250, 500). Each read was randomly shuffled in order to simulate synthetic negatives, which should not be classified into any protein family. Synthetic negatives were naively translated in 6 frames and searched against the SFams using RAPsearch2. Each read was classified according to its best hit across reading frames. (A-B) Empirical false positive rates at different bit-score cutoffs for different read lengths. Circles indicate optimal read-length specific cutoffs. (C) Empirical false positive rates versus E-values. Circles indicate optimal read-length specific cutoffs. (PDF) [file pcbi.1004573.s007.pdf]

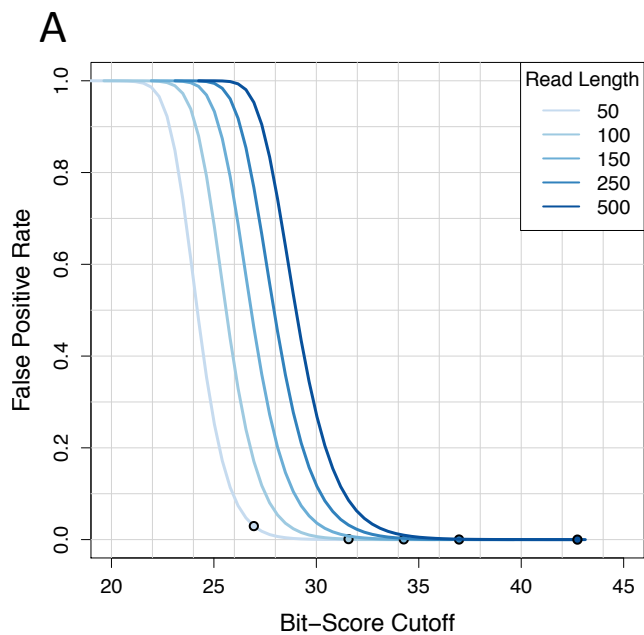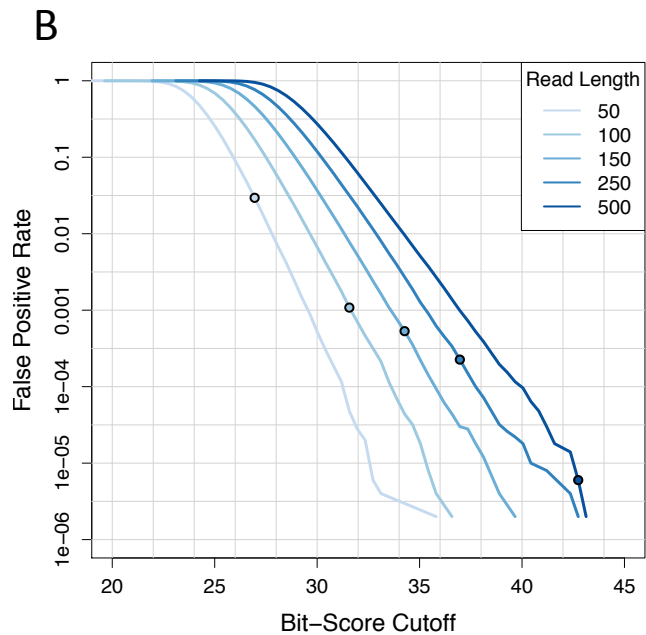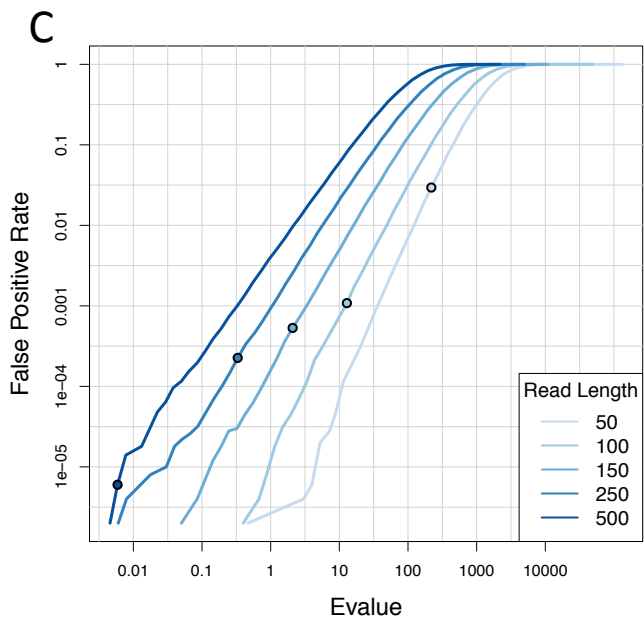

Supplement: S4 Fig — Effect of the sequencing error model on (A) read-length specific cutoffs and (B) relative abundance error. All simulated reads were 100 bp long from community 160319967-stool1. Error-free: reads simulated without sequencing error; Uniform: 1% uniform error rate; Illumina: exponential error model; 454: homopolymer error model; Sanger: linear error model ranging from 1% at the read start to 2% at the read end. (PDF) [file pcbi.1004573.s008.pdf]

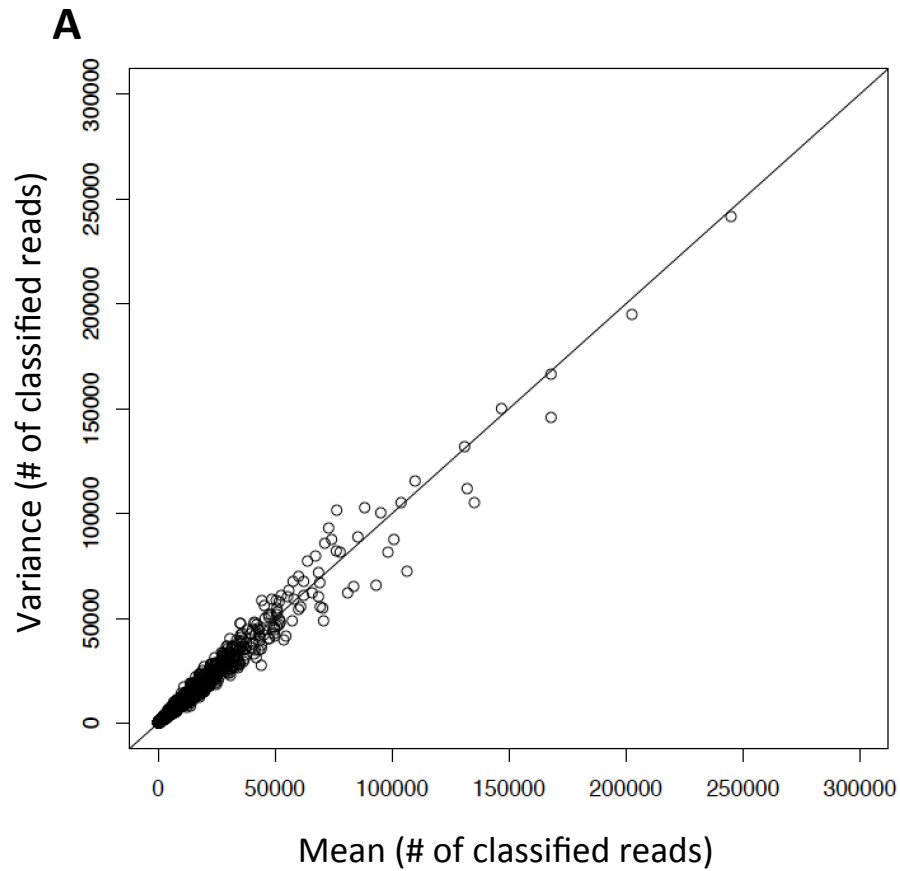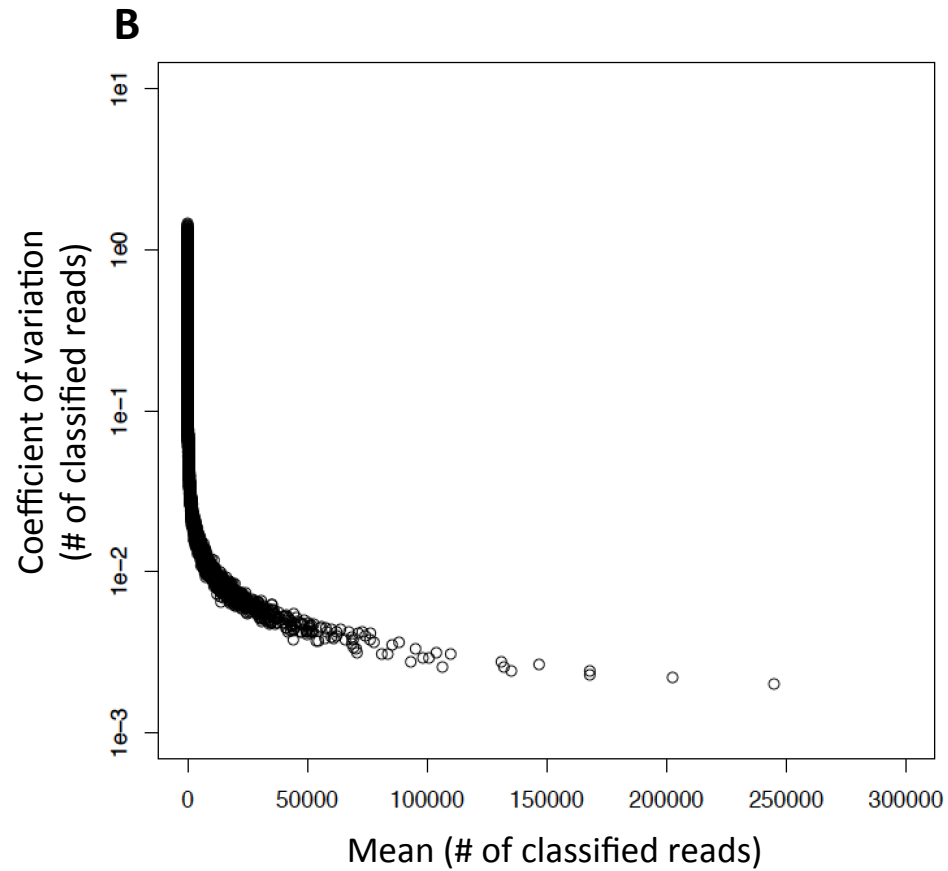

Supplement: S5 Fig — We used bootstrapping to estimate the variance of protein families as a function of the number of classified reads. Specifically, we resampled reads from the 100 million-read simulated metagenome, classified these reads into protein families, and computed abundance across families. We performed this procedure 100 times at each sequencing depth. a) In the absence of biological variation, protein families are Poisson distributed with mean equal to variance. b) The coefficient of variation (CV) of protein families decreases rapidly with increasing sequencing depth, and reaches an asymptote close to 1,000 reads and is sufficiently low at ~100 reads. For example, families with ~100 classified reads have a standard deviation of about 10 reads (CV = 0.10). This indicates it would take ~300,000 single-end Illumina reads to obtain a low-variance abundance estimate (CV ≤ 0.10) for a universally distributed single-copy gene present in a community with an average genome size of 3 Mb. (PDF) [file pcbi.1004573.s009.pdf]

KO Abundance All Families

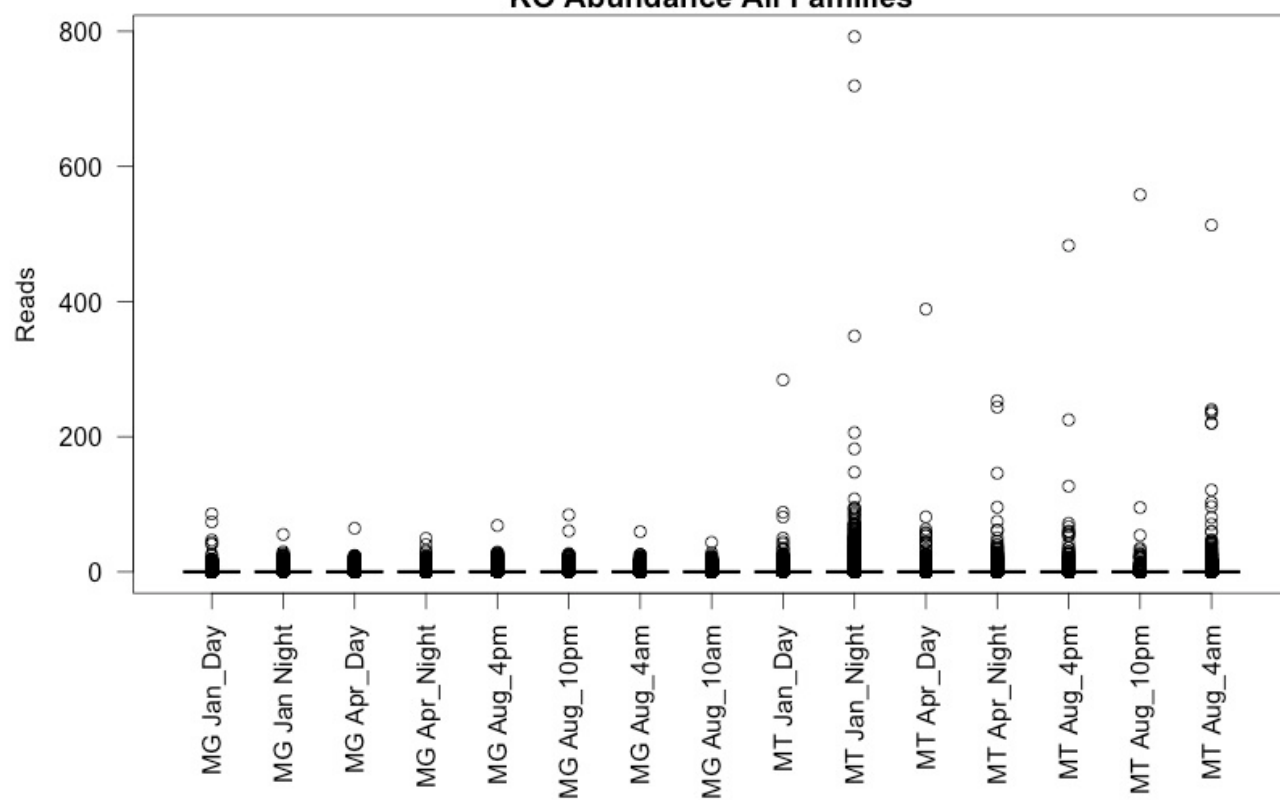

Supplement: S7 Fig — Boxplots for eight metagenomic and seven metatranscriptomic samples, which represent the distribution of the number of reads classified into each KO. Most KOs have a low classification rate, and metatranscriptomes (MT) are generally skewed towards having more highly abundant families than metagenomes (MG). (PDF) [file pcbi.1004573.s011.pdf]

## Metagenomes

## Metatranscriptomes

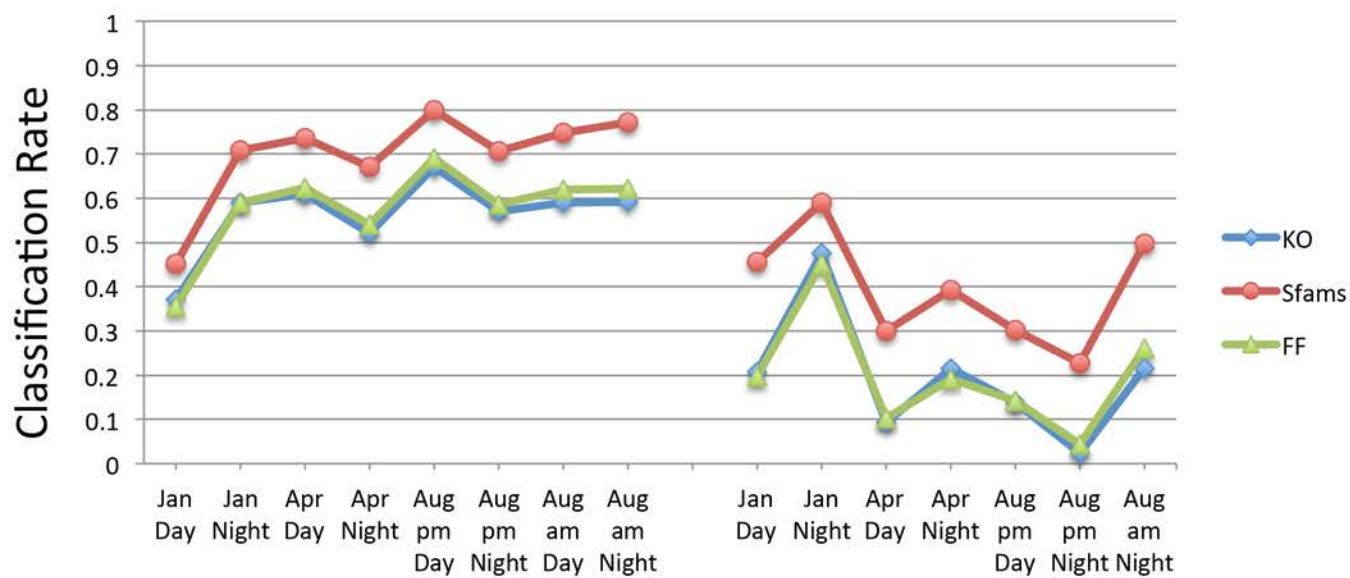

Sampling Timepoint

Supplement: S8 Fig — The rate at which sequences are classified into protein families (classification rate, y-axis) varies across databases (different colored lines; KEGG Orthology Groups (KO), Sifting Families (SFams), FigFams (FF)), data type (metagenomes (MG), metatranscriptomes (MT)), and season (as indicated along the x-axis). (PDF) [file pcbi.1004573.s012.pdf]

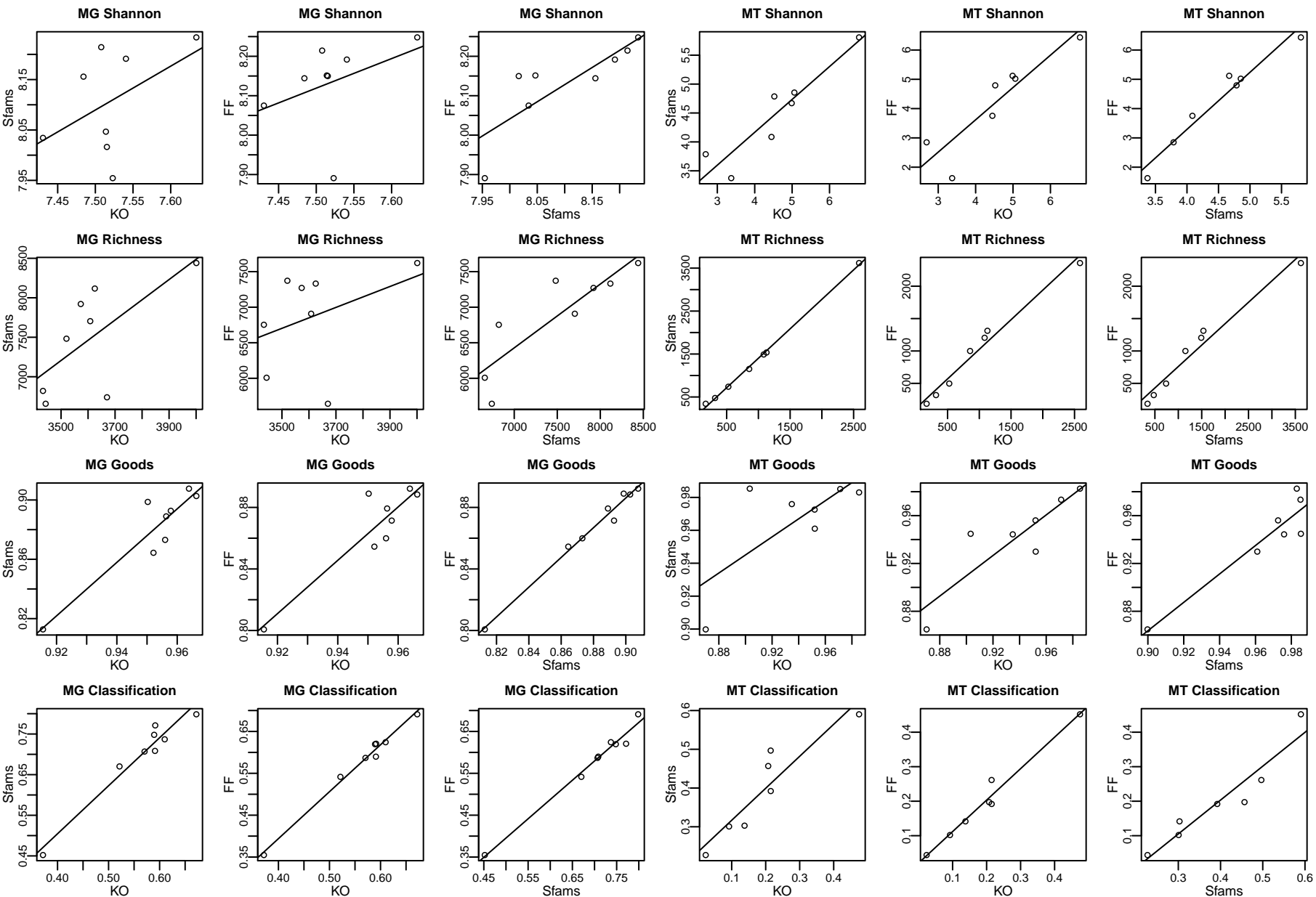

Supplement: S9 Fig — Pairwise scatterplots with regression lines comparing Shannon diversity, richness, Good’s coverage, and classification rate between the following databases: Kegg Orthology (KO) groups, Sfams, and FigFams (FF). Eight time points are plotted for metagenomic data (left) and seven time points for metatranscriptomic (right). (PDF) [file pcbi.1004573.s013.pdf]

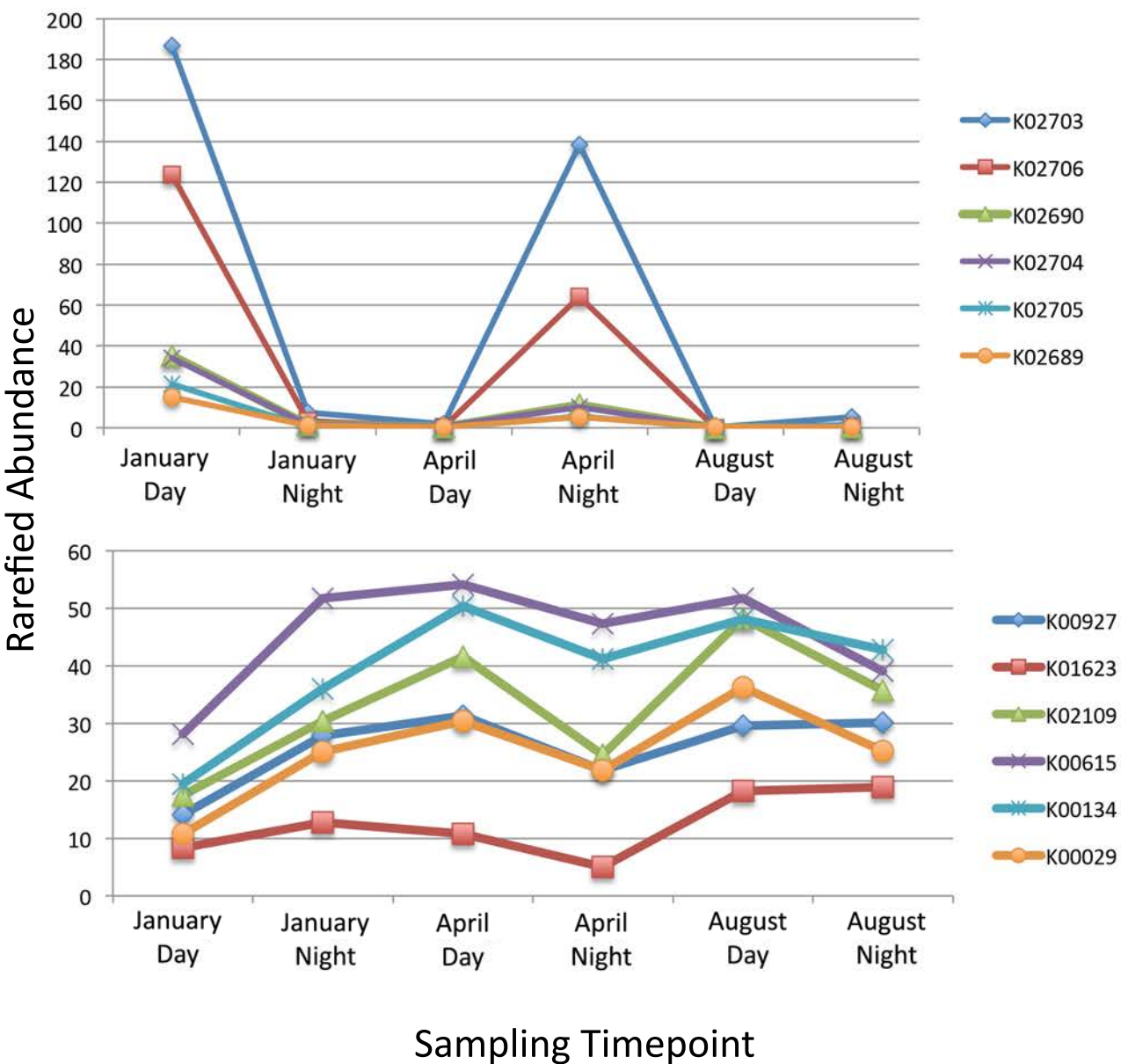

Supplement: S10 Fig — These plots illustrate change in the rarefied abundance (y-axis) across metagenomes (x-axis) of different KOs annotated as being involved in photosynthesis families. The upper plot represents the six KOs that show the same trends as Gilbert et al., which the lower plot represents six other KOs that show an opposite trend. (PDF) [file pcbi.1004573.s014.pdf]

**A**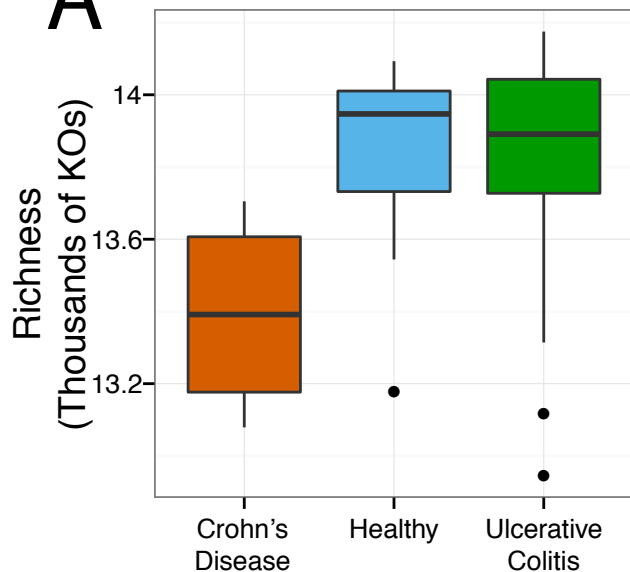**B**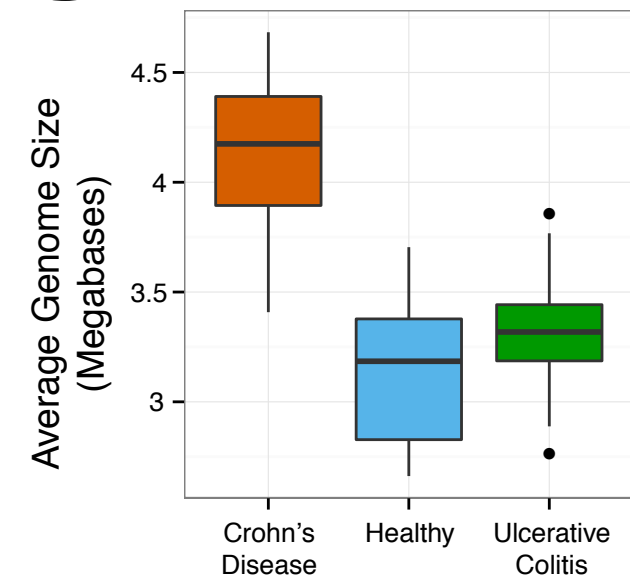**C**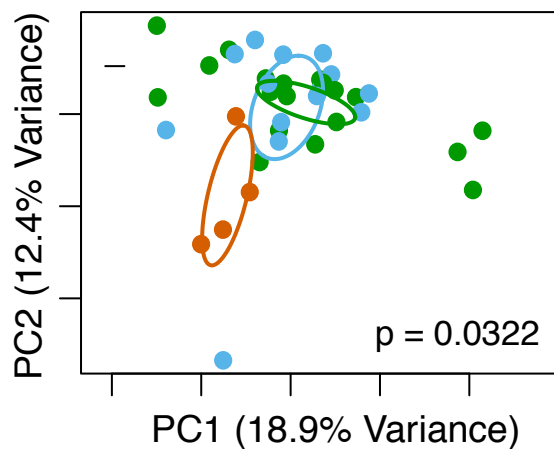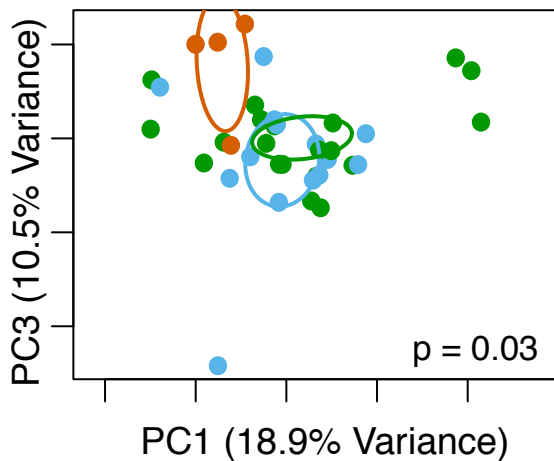

Supplement: S11 Fig — A smaller cohort of IBD patients and healthy controls (the MetaHIT cohort) were analyzed to assess the robustness of the patterns observed in the MGS cohort, which is described in the main text. (A) Boxplots illustrate that CD gut microbiomes exhibit lower protein family alpha-diversity than UC and H microbiomes (p < 0.05). Here, richness was calculated for each sample based on KO abundances, though the results are qualitatively consistent when SFams or MetaCyc families are quantified. (B) The average genome size of genomes comprising CD-associated microbiomes is significantly elevated relative to the other patient populations (p<0.05). (C) Principal components analysis (PCA) of protein family abundance profiles identifies differentiation in beta-diversity between CD (red) and non-CD populations. Here, PCA was conducted by zero centering and unit scaling KO abundances, though similar structure is identified using different protein family databases and PCA parameters. Ellipses represent 95% confidence intervals as quantified by the ordiellipse function in the R package vegan [45]. A permutational multivariate analysis of variance (adonis) quantified the reported p-values for the null hypothesis that the PCA axes are not different by group based on 1e4 permutations. (PDF) [file pcbi.1004573.s015.pdf]
