# Supplementary material for: Automated and Accurate Estimation of Gene Family Abundance from Shotgun Metagenomes
Source: PLoS Comput Biol. 2015 Nov 13;11(11):e1004573. doi: 10.1371/journal.pcbi.1004573 (PMC4643905; doi:10.1371/journal.pcbi.1004573)
Supplement: S6 Fig — Overall functional profiles for three databases calculated by ShotMAP. A-C Richness, Shannon entropy and Good’s coverage for KO, SFams and FIGfams over metagenomic samples for eight timepoints and metatranscriptomic samples for seven timepoints. Pearson’s rho pairwise across databases were (MG/MT): Richness KO:Sfams 0.67/0.99*; Sfams:FIGfams 0.87*/0.98*; KO:FIGfams 0.37/0.99*. Shannon KO:Sfams 0.47/0.93*;SFams:FIGfams 0.82*/0.89*;KO:FIGfams 0.39/0.93*.Good’s KO:SFams 0.92*/0.72; SFams:FIGfams 0.99*/0.94*;KO:FIGfams 0.91*/0.88*. *p<0.01. (PDF) [file pcbi.1004573.s010.pdf]

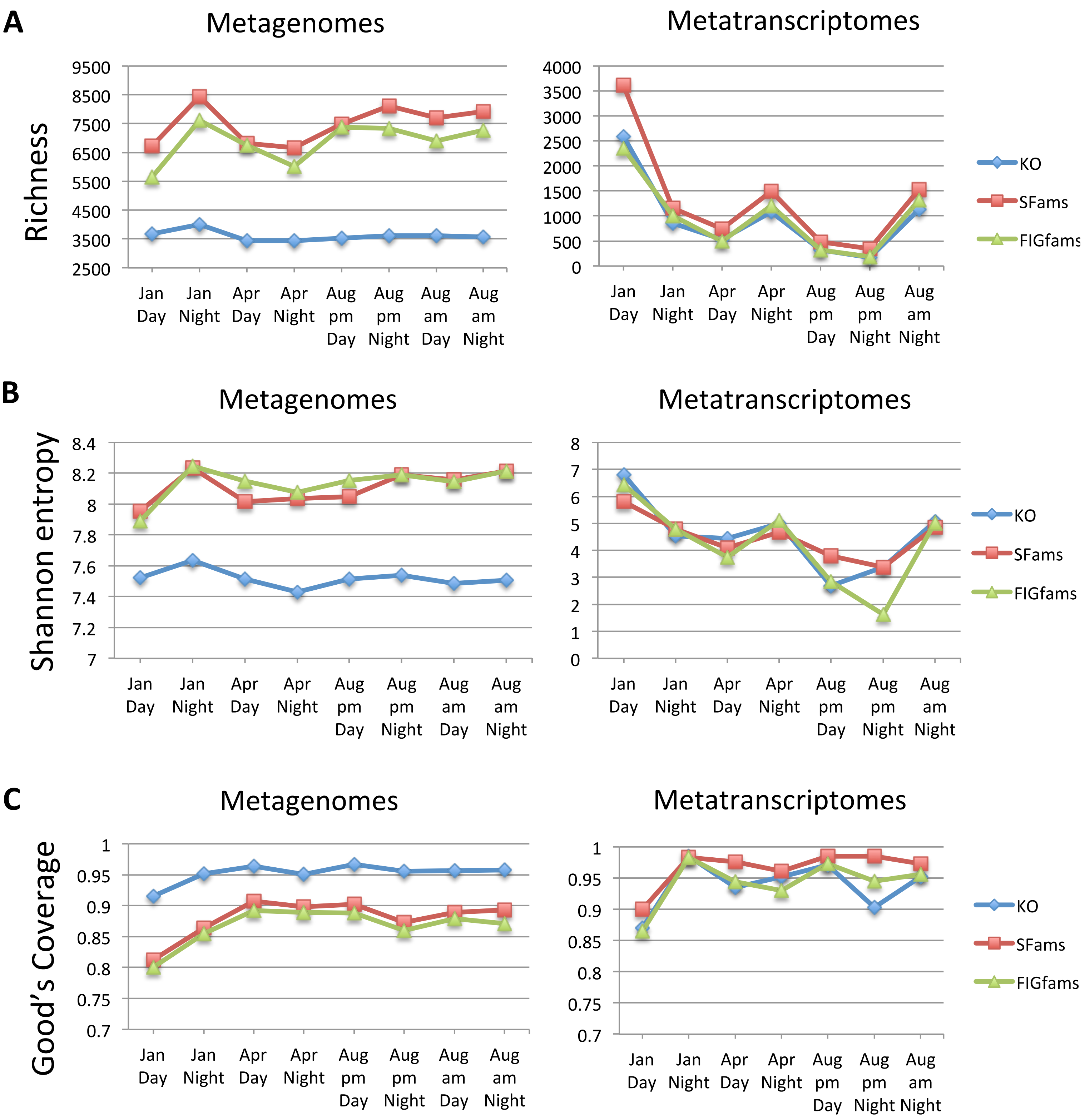

A
Metagenomes
Richness
9500
8500
7500
6500
5500
4500
3500
2500
Jan Day
Jan Night
Apr Day
Apr Night
Aug pm Day
Aug pm Night
Aug am Day
Aug am Night
Metatranscriptomes
4000
3500
3000
2500
2000
1500
1000
500
0
Jan Day
Jan Night
Apr Day
Apr Night
Aug pm Day
Aug pm Night
Aug am Night
KO
SFams
FIGfams
B
Metagenomes
Shannon entropy
8.4
8.2
8
7.8
7.6
7.4
7.2
7
Jan Day
Jan Night
Apr Day
Apr Night
Aug pm Day
Aug pm Night
Aug am Day
Aug am Night
Metatranscriptomes
8
7
6
5
4
3
2
1
0
Jan Day
Jan Night
Apr Day
Apr Night
Aug pm Day
Aug pm Night
Aug am Night
KO
SFams
FIGfams
C
Metagenomes
Good's Coverage
1
0.95
0.9
0.85
0.8
0.75
0.7
Jan Day
Jan Night
Apr Day
Apr Night
Aug pm Day
Aug pm Night
Aug am Day
Aug am Night
Metatranscriptomes
1
0.95
0.9
0.85
0.8
0.75
0.7
Jan Day
Jan Night
Apr Day
Apr Night
Aug pm Day
Aug pm Night
Aug am Night
KO
SFams
FIGfams
