# Supplementary material for: Automated and Accurate Estimation of Gene Family Abundance from Shotgun Metagenomes
Source: PLoS Comput Biol. 2015 Nov 13;11(11):e1004573. doi: 10.1371/journal.pcbi.1004573 (PMC4643905; doi:10.1371/journal.pcbi.1004573)
Supplement: S1 Text — (DOCX) [file pcbi.1004573.s016.docx]

**Description of ShotMAP**

ShotMAP is a computational workflow that automates the functional annotation of metagenomic data and the statistical comparison of functional profiles across metagenomes. The workflow takes as input a one or more quality-controlled metagenome sequence files (i.e., samples), each of which corresponds to a distinct sample, and a protein family database (i.e., reference database), which serves as the reference for annotating metagenomic sequences. Notably, ShotMAP is agnostic to how the data is preprocessed (e.g., assembled or not), the specific reference database, though specific parameters and settings may need to be adjusted to ensure maximum annotation accuracy [Main Text]. Additionally, ShotMAP can process either metagenomic or metatranscriptomic sequences.

ShotMAP implements the core metagenome annotation procedure described in the Main Text, but provides various analytical options to accommodate different user needs. By default, ShotMAP implements parameters based on the input data, such as read length. ShotMAP begins by predicting coding sequences in each sample. While it implements prodigal by default on sequences that are 70 bp in length, users can specify additional prediction methods including 6FT (with or without length filtering), which is the default for shorter sequences. Each sample’s predicted peptides are then compared to an index search database, which ShotMAP assembles from the reference database. Various alignment algorithms are used to compare sequences to the database, including BLAST, various fast-blast tools like RAPsearch2, and HMMER v3. Various user-defined thresholds (e.g., alignment score, e-value, coverage) are then used to classify sample sequences into a single reference database families based on their predicted peptides’ best hit. ShotMAP then evaluates the distribution of classified sequences across families to profile reference family abundance within each sample. Here, abundance can be estimated using either the number of sequences classified into a family (i.e., counts), the aligned coverage of the average family member sequence by predicted peptides (i.e., coverage), and the family length normalized versions of these abundances. ShotMAP then uses MicrobeCensus to calculate and normalize abundances by the average genome size of the community. Family abundance profiles are then used to characterize the functional diversity of each sample (e.g., richness, Shannon entropy), statistically compare the diversity profiles across samples, quantify relationships between functional diversity and sample covariates (i.e., metadata), identify families that stratify samples grouped by metadata properties, and quantify associations between family abundance and metadata variables. ShotMAP uses robust, non-parametric statistical tests for these comparative analyses (e.g., Wilcoxon tests, kruskal-wallis tests, kendall’s tau). Many of these statistical comparisons require that sequence depth be similarly uniform across samples, so ShotMAP implements one of two way of rarefying samples: (1) sequences are subsampled and abundances are recalculated for each sample based only on the rarified data or (2) users predetermine the number of sequences that are to be processed for each sample.

ShotMAP takes several steps to increase compute throughput and reduce disc footprint. First, ShotMAP parallelizes the annotation process. For example, sample and reference database sequences (or HMMs) are partitioned into files that can be independently compared on distinct processors. Additionally, while ShotMAP’s default state is to run on a multi-core, lab server, it is configured to automatically manage the communication and transport of data with an SGE configured cloud computer to improve parallelization. Additionally, ShotMAP can optionally communicate with a MySQL database for improved management and indexing of the data. Finally, ShotMAP works in compressed sequence space where possible to reduce the filesize footprint needed to process a sample.

ShotMAP is written in Perl, python and R, and leverages a variety of open source, third party software tools. The source code can be obtained from <https://github.com/sharpton/shotmap>.
