# Supplementary material for: Automated and Accurate Estimation of Gene Family Abundance from Shotgun Metagenomes
Source: PLoS Comput Biol. 2015 Nov 13;11(11):e1004573. doi: 10.1371/journal.pcbi.1004573 (PMC4643905; doi:10.1371/journal.pcbi.1004573)
Supplement: S2 Text — (DOCX) [file pcbi.1004573.s017.docx]

**L4 Data Analysis Supplemental Methods**

L4 shotgun sequencing datasets of DNA for eight samples and mRNA for seven samples were downloaded from the MG-RAST website (<http://metagenomics.anl.gov/metagenomics.cgi?page=MetagenomeProject&project=109>). The pre-filtered datasets ranged from 267,833 to 631,084 reads for the DNA samples and 47,406 to 80,615 reads for the RNA samples. Datasets were pre-filtered for quality and to remove rRNA sequences from the metatranscriptomic datasets. We used ShotMAP to functionally annotate the datasets using three databases: the FIGfams database from the Fellowship for the Interpretation of Genomes (Release 12, 2009. http://www.nmpdr.org/FIG/wiki/view.cgi/FIG/FigFam) that Gilbert et al. used, as well as the KEGG Orthology (KO, downloaded June 25, 2013) and the Sifting Families (SFams v 1.0) databases.  The FIGfam database consists of 1,422,349 proteins comprising 213,098 families, the KOs are comprised of 4,078,985 proteins across 18,416 families, and SFams have 6,098,117 proteins distributed in 345,641 families. To facilitate comparisons across samples, the eight metagenomic datasets were rarefied to 267,833 reads (the size of the smallest metagenomic dataset) and the seven metatranscriptomic datasets were rarefied to 47,406 reads (the size of the smallest metatranscriptomic dataset). Reads were translated in all six reading frames and split on stop codons, and then ORF’s having a length of at least 15 amino acids and a RAPSearch bit score of at least 35 were classified in each of the three databases according to their best protein sequence hit within each database.  Family abundance was then calculated as the number of residues of the translated metagenomic read that align to the target protein from the family normalized by the length of the average family member. Raw abundances for the metagenomic datasets were then corrected for average genome size using MicrobeCensus. For each database, we calculated physiological richness (the number of families observed in a sample), Good’s coverage (1 – the number of families with a single hit/number of classified reads for a sample), Shannon entropy, and the overall classification rate for the 15 samples with respect to each of the three functional protein family databases (Supplemental Figure 7 A, B, and C).

**Challenges associated with the analysis of low-abundance gene families**

We observed several noteworthy results regarding low-abundance gene families in the L4 data. First, most of the observed gene families in the L4 metagenomes and metatranscriptomes have a low absolute abundance (e.g., only 6% of families had over 100 reads in KEGG and only 1% with FIGfams). This long-tailed skew in the gene family rank-abundance distribution is common in the analysis of metagenomes and indicates that, for most families, differences across samples will be highly subject to stochastic effects. As a result, caution is warranted when interpreting seasonal or diel trends for individual families or potentially even pathways. Second, the proportion of reads classified into gene families varies substantially between samples (S12 Figure). Care should thus be taken when assessing a family’s inter-sample relative abundance distribution, since this measurement is highly dependent on classification rate: low classification rates artificially inflate relative abundance estimates for observed gene families. We believe this issue explains why we find lower functional diversity in January day metagenomes than was originally reported with the data; this sample has the lowest classification rate of all metagenomes. Finally, gene families supported by a single read also play an important role at the level of community diversity. For example, we observed higher richness in January day compared to January night metatranscriptomes with all three databases, but much of this was due to families consisting of a single read. Based on our simulations, we hypothesize that this diversity caused by the large number of low abundance families in daytime samples is largely real (i.e., low predicted error rates), despite conflicting with previous studies that found elevated richness in night-time samples (e.g., (1),(2)). ShotMAP provides the computational tools and summary statistics necessary to explore and address these sample-specific factors that may confound intersample comparisons of distinct gene families.

**References**

1. Poretsky RS, Hewson I, Sun S, Allen AE, Zehr JP, Moran MA. Comparative day/night metatranscriptomic analysis of microbial communities in the North Pacific subtropical gyre. Environ Microbiol [Internet]. 2009 Jun [cited 2015 Feb 28];11(6):1358–75. Available from: http://www.ncbi.nlm.nih.gov/pubmed/19207571

2. Gilbert JA, Steele JA, Caporaso JG, Steinbrück L, Reeder J, Temperton B, et al. Defining seasonal marine microbial community dynamics. ISME J [Internet]. 2012 Feb [cited 2014 Mar 21];6(2):298–308. Available from: http://www.pubmedcentral.nih.gov/articlerender.fcgi?artid=3260500&tool=pmcentrez&rendertype=abstract
