# Supplementary material for: Automated and Accurate Estimation of Gene Family Abundance from Shotgun Metagenomes
Source: PLoS Comput Biol. 2015 Nov 13;11(11):e1004573. doi: 10.1371/journal.pcbi.1004573 (PMC4643905; doi:10.1371/journal.pcbi.1004573)

**The Impact of Filtering Families in the MetaHIT data Family Filtering Supplemental Methods**

In order to improve our ability to detect true positive associations between clinical variables (e.g., age, body mass index (BMI), inflammatory bowel disease status (none, ulcerative colitis, and Crohn's disease), and gender), we explored how independent filtering on gene families impacted discovery. Here, we focused on the 39 high-quality metagenomes produced by Qin et al. that were described in the main text. We used ShotMAP to annotate 20M reads from each of the MetaHIT samples using three different databases: KO, MetaCyc, and SFam databases. We then tested how filtering gene families based on the following statistics affected discovery: mean, variance, coefficient of variation (computed as the standard deviation over the mean, scaled by 1 + 1/(4n)), and the number of zero values (i.e., the number of samples in which that protein family was not detected by ShotMAP). For the first three statistics, genes above a particular threshold were retained, while for the last, genes below a particular threshold were retained.

Filtering based on coefficient of variation did not appear to yield any improvements in the number of significant discoveries (see Figure below). Filtering by mean and variance, however, did improve discoveries in each database, which is consistent with previous applications of this technique. We could not identify a threshold that performed optimally for all three annotation spaces. In contrast, filtering by the number of zero values led to monotonic improvements across all three databases, such that keeping only fully-observed gene families (i.e., with no zero values) maximized the number of discoveries in each database at a q-value cutoff of 0.1.

A similar analysis with the MGS cohort data, which were only annotated using KOs, did not reveal a consistent result, as filtering based on fully-observed gene families decreased the number of discoveries somewhat (3,441 vs. 4,926 with no filtering). These results indicate that the degree of improvement that can be obtained via gene family filtering may depend on specific characteristics of the dataset under consideration.

***Figure: Impact of thresholding hypothesis tests of family abundance based on the occurrence of a family across samples***

*Effect of different filtering procedures on the number of associations made between gene families and phenotypes. Phenotypes are listed in columns ("ibd" refers to a binary phenotype where both UC and CD cases are compared against controls; "ibdC" separates UC, CD, and controls). Statistics used for filtering are listed in rows ("m" = mean, "v" = variance, "cv" = coefficient of variation, and "z" = number of zeros). The x-axis gives the cutoff as a percent of the most stringent cutoff possible (i.e., from left to right, fewer gene families were retained). The different databases are shown in color (in red, "KO" = KEGG; in green, "SFAM" = SFams; in blue, "MC" = MetaCyc). Filtering out gene families that contained zeros yields the most consistent improvement across gene family databases; also, ibdC (i.e., UC vs. CD vs. control) yielded the most discoveries regardless of the method of filtering.*


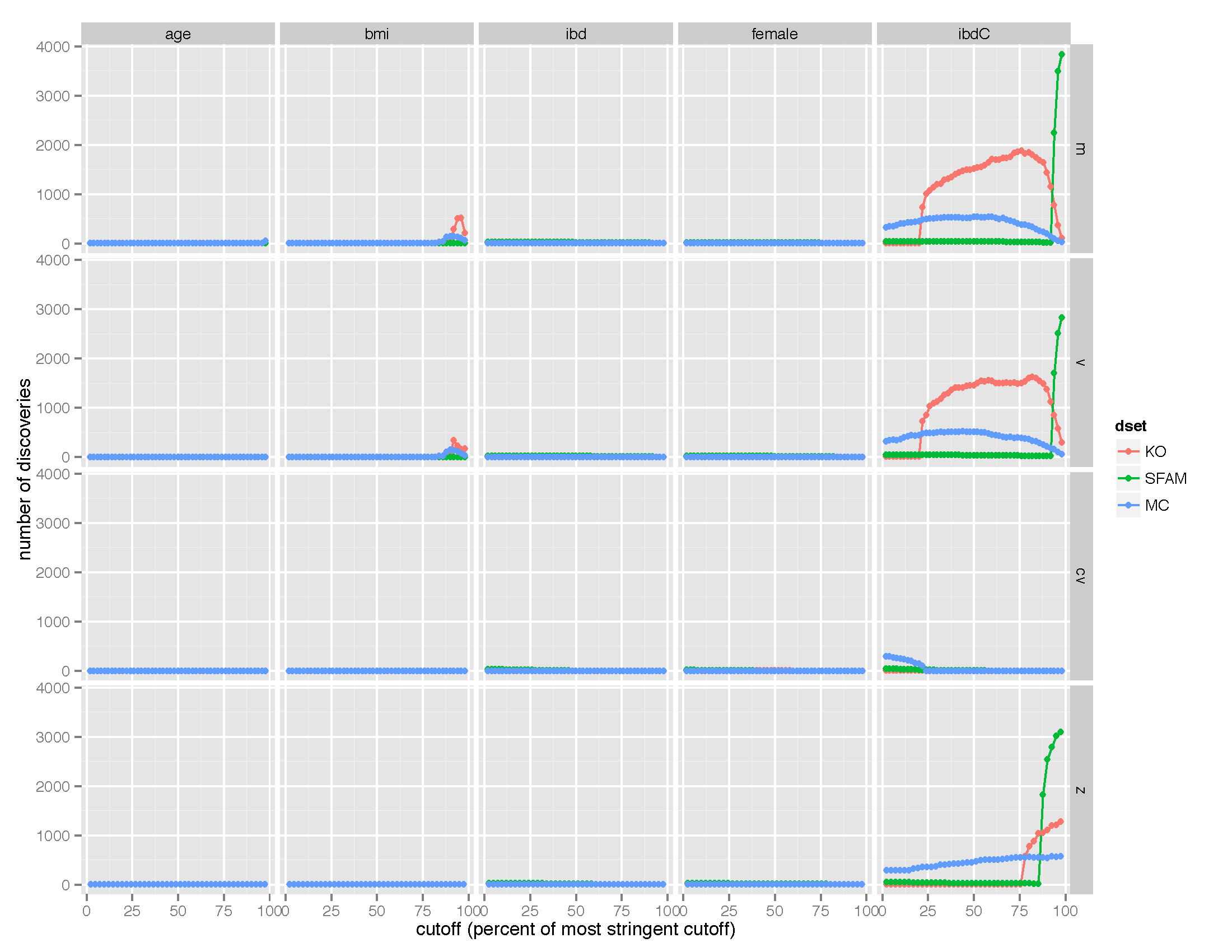

Supplement: S3 Text — (DOCX) [file pcbi.1004573.s018.docx]
